# Supplementary material for: Alcohol consumption among university students in ASEAN countries: A systematic review and meta-analysis
Source: Glob Ment Health (Camb). 2025 Jun 20;12:e69. doi: 10.1017/gmh.2025.10027 (PMC12231523; doi:10.1017/gmh.2025.10027)
Supplement: Kejriwal supplementary material [file S2054425125100277sup001.pdf]

*Cambridge Prisms*

**www.cambridge.org**

**Review**

# Supplementary Material

Mayank Kejriwal*

University of Southern California, Los Angeles, CA 90292, United States of America

*Author for correspondence. Email: keriwal@isi.edu

## Appendix A: Detailed Database-specific Search Strategies

### *PubMed*

1. (alcoho* OR bing* OR drink*) OR ("Alcoholism"[Mesh])
   753,245 found
2. ((socio?demo*) OR (demograp*) OR (income) OR (ethnic*) OR (religio*) OR (socio?econ*))
   1,070,774 found
3. ("Sociodemographic Factors"[Mesh]) OR ("Socioeconomic Factors"[Mesh])
   526,342 found
4. (colleg* OR adoles* OR universi* OR studen* OR institut* OR "higher education" OR "young adult")
   23,202,255 found
5. "Underage Drinking"[Mesh]
   1,293 found
6. "Adolescent"[Mesh]
   2,267,809 found
7. "Alcohol Drinking in College"[Mesh]
   1,057 found
8. "College fraternities and sororities"[Mesh]
   38 found
9. ("Association of Southeast Asian Nations" OR "asean" OR "burma" OR burmes* OR cambodi* OR indones* OR "laos" OR malay* OR "malay" OR philippi* OR filipp* OR "thai" OR thai* OR vietna*)
   397,806 found
10. ("Asian people"[Mesh])
    92,381 found
11. "Myanmar"[Mesh] OR "Cambodia"[Mesh] OR "Indonesia"[Mesh] OR "Laos"[Mesh] OR "Malaysia"[Mesh] OR "Philippines"[Mesh] OR "Thailand"[Mesh] OR "Vietnam"[Mesh]
    95,865 found

**Final query:** ((((alcoho* OR bing* OR drink*) OR ("Alcoholism"[Mesh])) AND (colleg* OR adoles* OR universi* OR studen* OR institut* OR "higher education" OR "young adult" OR "Adolescent"[Mesh] OR "College fraternities and sororities"[Mesh])) OR ("Underage Drinking"[Mesh] OR "Alcohol Drinking in College"[Mesh])) AND (((socio?demo*) OR (demograp*) OR (income) OR (ethnic*) OR (religio*) OR (socio?econ*)) OR ("Sociodemographic Factors"[Mesh]) OR ("Socioeconomic Factors"[Mesh])) AND (("Association of Southeast Asian Nations" OR "asean" OR "burma" OR burmes* OR cambodi* OR indones* OR "laos" OR malay* OR "malay" OR philippi* OR filipp* OR "thai" OR thai* OR vietna*) OR ("Asian people"[Mesh]) OR ("Myanmar"[Mesh] OR "Cambodia"[Mesh] OR "Indonesia"[Mesh] OR "Laos"[Mesh] OR "Malaysia"[Mesh] OR "Philippines"[Mesh] OR "Thailand"[Mesh] OR "Vietnam"[Mesh]))

CAMBRIDGE
UNIVERSITY PRESS

### *Global Index Medicus*

1. (alcoho*) AND ( collection_gim:("WPRIM" OR "IMSEAR"))
   16,534 found
2. (binge*) OR (drink*) AND ( collection_gim:("WPRIM" OR "IMSEAR"))
   9,452 found
3. (mh:c25.775.100.250* OR mh:f03.900.100.350*) AND ( collection_gim:("WPRIM" OR "IMSEAR"))
   606 found
4. ((socio?demo*) OR (demograp*) OR (income) OR (ethnic*) OR (religio*) OR (socio?econ*)) AND ( collection_gim:("WPRIM" OR "IMSEAR"))
   35,125 found
5. (mh:I01.880.853.965* OR mh:I01.880.853.996* OR mh:N01.824* OR mh:SP2.070.315.420.566* OR mh:SP3.311.900.686.409* OR mh:SP3.522.233*) AND ( collection_gim:("WPRIM" OR "IMSEAR"))
   8,454 found
6. (colleg* OR adoles* OR universi* OR studen* OR institut* OR "higher education" OR "young adult") AND ( collection_gim:("WPRIM" OR "IMSEAR"))
   192,659 found
7. (mh:F01.145.022.750* OR mh:F01.145.317.269.875* OR mh:I01.880.735.878*) AND ( collection_gim:("WPRIM" OR "IMSEAR"))
   2 found
8. (mh:M01.060.057*) AND (collection_gim:("WPRIM" OR "IMSEAR"))
   40,853 found
9. (mh:F01.145.317.269.625) AND (collection_gim:("WPRIM" OR "IMSEAR"))
   3 found
10. (mh:N03.540.162*) AND (collection_gim:("WPRIM" OR "IMSEAR"))
    0 found
11. ("Association of Southeast Asian Nations" OR "asean" OR "burma" OR burmes* OR cambodi* OR indones* OR "laos" OR malay* OR "malay" OR philippi* OR filipp* OR "thai" OR thai* OR vietna*) AND ( collection_gim:("WPRIM" OR "IMSEAR"))
    28,267 found
12. (mh: M01.686.330*) AND (collection_gim:("WPRIM" OR "IMSEAR"))
    0 found

**Final query:** (((((alcoho*)) OR ((binge*) OR (drink*)) OR (mh:c25.775.100.250* OR mh:f03.900.100.350*)) AND ((colleg* OR adoles* OR universi* OR studen* OR institut* OR "higher education" OR "young adult") OR (mh:M01.060.057*) OR (mh:N03.540.162*))) OR ((mh:F01.145.022.750* OR mh:F01.145.317.269.875* OR mh:I01.880.735.878*) OR (mh:F01.145.317.269.625))) AND (((socio?demo*) OR (demograp*) OR (income) OR (ethnic*) OR (religio*) OR (socio?econ*)) OR (mh:I01.880.853.965* OR mh:I01.880.853.996* OR mh:N01.824* OR mh:SP2.070.315.420.566* OR mh:SP3.311.900.686.409* OR mh:SP3.522.233*)) AND (("Association of Southeast Asian Nations" OR "asean" OR "burma" OR burmes* OR cambodi* OR indones* OR "laos" OR malay* OR "malay" OR philippi* OR filipp* OR "thai" OR thai* OR vietna*) OR (mh: M01.686.330*)) AND (collection_gim:("WPRIM" OR "IMSEAR"))

### *Global Health*

1. (alcoho* or (binge adj3 drink*)).ti,ab,kw.
2. (socio?demo* or demograp* or income or ethnic* or religio*).ti,ab,kw.
3. socio?econ*.ti,ab,kw.
4. (colleg* or adoles* or universi* or student* or (young adj2 adult) or (high* adj2 educ* or institu*).ti,ab,kw.
5. ("Association of Southeast Asian Nations" or ASEAN or burma or burmes* or cambodi* or Indones* or Laos or Malay* or Malay or Philippi* or Filipp* or Thai or thai* or vietna*).ti,ab,kw.
6. exp "drinking"/ use cagz or exp "alcoholic beverages"/ use cagz
7. exp ”alcoholism"/ use cagz or exp "alcohol intake"/ use cagz
8. exp ”socioeconomic status"/ use cagz or exp "demography"/ use cagz or exp "risk factors"/ use cagz or exp "education"/ use cagz or exp "ethnicity"/ use cagz
9. 1 or 6 or 7
10. 2 or 3 or 8
11. exp "college students"/ use cagz or exp "students"/ use cagz or exp "adolescents"/ use cagz
12. exp "South East Asia"/ use cagz or exp "Thailand"/ use cagz or exp "Indonesia"/ use cagz or exp "Philippines"/ use cagz or exp "Asia"/ use cagz or exp ”Malaysia"/ use cagz
13. 4 or 11
14. 5 or 12
15. 9 and 10 and 13 and 14

### *Garuda Rujukan Digital*

As the search interface was very basic, we searched on “alcoholism” (96 documents) and “alcohol addiction” (65 documents); then filtered manually. Many articles are non-English and excluded. Four potentially relevant publications were identified and retained.

### *Web of Science*

1. ALL=(alcoho*)
   815,052 found
2. TS=( binge NEAR/3 drink* ) OR TI=( binge NEAR/3 drink* ) OR AB=( binge NEAR/3 drink* )
   11,977 found
3. ALL=( socio?demo* OR demograp* OR income OR ethnic* OR religio* OR socio?econ*)
   1,564,089 found
4. ALL=( colleg* OR adoles* OR universi* OR studen* OR institut*) OR TS=(young NEAR/2 adult) OR TS=(high* NEAR/2 educ* ) OR TI=(young NEAR/2 adult) OR TI=(high* NEAR/2 educ* ) OR AB=(young NEAR/2 adult) OR AB=(high* NEAR/2 educ* )
   39,708,789 found

5. ALL= ( "Association of Southeast Asian Nations" OR asean OR burma OR burmes* OR cambodi* OR indones* OR laos OR malay* OR malay OR philippi* OR filipp* OR thai OR thai* OR vietna* )
   1,443,869 found
6. (1 OR 2) AND 3 AND 4 AND 5
   1066 found

### *Scopus*

1. TITLE-ABS-KEY ( alcoho* OR ( binge W/3 drink* ))
   1,107,406 documents found
2. TITLE-ABS-KEY ( socio?demo* OR demograp* OR income OR ethnic* OR religio* OR socio?econ*)
   2,144,365 documents found
3. TITLE-ABS-KEY ( colleg* OR adoles* OR universi* OR studen* OR ( young W/2 adult ) OR ( high* W/2 educ* ) OR institut* )
   8,881,510 documents found
4. TITLE-ABS-KEY ( "Association of Southeast Asian Nations" OR asean OR burma OR burmes* OR cambodi* OR indones* OR laos OR malay* OR malay OR philippi* OR filipp* OR thai OR thai* OR vietna* )
   660,740 documents found
5. (TITLE-ABS-KEY ( "Association of Southeast Asian Nations" OR asean OR burma OR burmes* OR cambodi* OR indones* OR laos OR malay* OR malay OR philippi* OR filipp* OR thai OR thai* OR vietna* )) AND (TITLE-ABS-KEY ( colleg* OR adoles* OR universi* OR studen* OR ( young W/2 adult ) OR ( high* W/2 educ* ) OR institut* )) AND (TITLE-ABS-KEY ( socio?demo* OR demograp* OR income OR ethnic* OR religio* OR socio?econ*)) AND (TITLE-ABS-KEY ( alcoho* OR ( binge W/3 drink* )))
   636 documents found

### *Medline*

1. (alcoho* or (binge adj3 drink*)).mp.
2. (socio?demo* or demograp* or income or ethnic* or religio*).mp.
3. socio?econ*.mp.
4. (colleg* or adoles* or universi* or student* or (young adj2 adult) or (high* adj2 educ* or institu*).mp.
5. ("Association of Southeast Asian Nations" or ASEAN or burma or burmes* or cambodi* or Indones* or Laos or Malay* or Malay or Philippi* or Filipp* or Thai or thai* or vietna*).mp.
6. exp "Underage Drinking"/ use medall or exp "Alcoholic Drinking"/ use medall or exp ”Alcohol Beverages"/ use medall
7. exp "Alcoholism"/ use medall or exp "Alcohol Drinking"/ use medall
8. exp ”Socioeconomic Factors"/ use medall or exp "Sociodemographic Factors"/ use medall
9. 1 or 6 or 7
10. 2 or 3 or 8
11. exp "Students"/ use medall or exp "Universities"/ use medall or exp "Young Adult"/ use medall
12. exp "Asia, Southeastern"/ use medall or exp "Thailand"/ use medall or exp "Indonesia"/ use medall or exp "Philippines"/ use medall or exp ”Malaysia"/ use medall
13. 4 or 11
14. 5 or 12
15. 9 and 10 and 13 and 14

### *Embase*

1. (alcoho* or (binge adj3 drink")).mp.
2. (socio?demo* or demograp* or income or ethnic* or religio*).mp. [mp=title, abstract, heading word, drug trade name, original title, device manufacturer, drug manufacturer, device trade name, keyword heading word, floating subheading word, candidate term word]
3. socio?econ*.mp.
4. (colleg* or adoles* or universi* or student* or (young adj2 adult) or (high* adj2 educ*) or institut*).mp.
5. ("Association of Southeast Asian Nations" or ASEAN or burma or burmes* or cambodi* or Indones* or Laos or Malay* or Malay or Philippi* or Filipp* or Thai or thai* or vietna*).mp. [mp=title, abstract, heading word, drug trade name, original title, device manufacturer, drug manufacturer, device trade name, keyword heading word, floating subheading word, candidate term word]
6. exp "underage drinking"/ or exp "drinking behavior"/ use emczd or exp "alcohol abuse"/ use emczd or exp "alcohol consumption"/ use emczd or exp "alcohol intoxication"/ use emczd or exp "Alcohol Use Disorders Identification Test"/ use emczd or exp "alcohol rehabilitation"/ use emczd or exp "alcohol urge questionnaire"/ use emczd or exp "alcohol withdrawal seizure"/ use emczd
7. exp "alcoholism"/ use emczd or exp "experimental alcoholism"/ use emczd
8. exp "sociodemographics"/ use emczd or exp "socioeconomics"/ use emczd
9. 1 or 6 or 7
10. 2 or 3 or 8
11. exp "college student"/ use emczd or exp "adolescent"/ use emczd
12. exp "Southeast Asia"/ use emczd or exp "Thailand"/ use emczd or exp "Indonesia"/ use emczd or exp "Philippines"/ use emczd or exp "Asia"/ use emczd or exp "Malaysia"/ use emczd
13. 4 or 11
14. 5 or 12
15. 9 and 10 and 13 and 14

### *Cochrane*

1. MeSH descriptor: [Alcoholism] explode all trees
   5,130 documents found
2. MeSH descriptor: [Sociodemographic Factors] explode all trees
   15 documents found
3. MeSH descriptor: [Socioeconomic Factors] explode all

trees
14,434 documents found
4. ((alcoho*) OR (binge NEAR/3 drink*)):ti,ab,kw
40,717 documents found
5. 1 OR 4
40,717 documents found
6. (socio?demo* OR demograp* OR income OR ethnic* OR religio*):ti,ab,kw
*(word variations have been searched)*
86,222 documents found
7. (socio?econ*):ti,ab,kw
*(word variations have been searched)*
13,040 documents found
8. 6 OR 7 OR 2 OR 3
100,904 documents found
9. (colleg* OR adoles* OR universi* OR student* OR (young NEAR/2 adult) OR (high* NEAR/2 educ*) OR (institut*)):ti,ab,kw
*(word variations have been searched)*
397,256 documents found
10. ("Association of Southeast Asian Nations" OR ASEAN OR burma OR burmes* OR cambodi* OR Indones* OR Laos OR Malay* OR Philippi* OR Filipp* OR Thai OR thai* OR vietna*):ti,ab,kw
*(word variations have been searched)*
25,623 documents found
11. MeSH descriptor: [Underage Drinking] explode all trees
127 documents found
12. MeSH descriptor: [Adolescent] this term only
137,774 documents found
13. MeSH descriptor: [Alcohol Drinking in College] explode all trees
167 documents found
14. MeSH descriptor: [College Fraternities and Sororities] explode all trees
5 documents found
15. MeSH descriptor: [Athletes] explode all trees
1,773 documents found
16. MeSH descriptor: [Asian] explode all trees
342 documents found
17. 15 OR 14 OR 12 OR 9
397,775 documents found
18. 16 OR 10
25,908 documents found
19. 8 AND ((5 AND 17) OR (11 OR 13)) AND 18
48 documents found

## Appendix B: Quality Appraisal of Selected Studies using AXIS

Table 1 provides quality appraisal of each study included in the review using the AXIS instrument. The specific questions underlying the question IDs in the table are reproduced in Table 2.

## Appendix C: Inclusion criteria based on PICOST framework

Details governing each of the PICOST categories that were used for developing the inclusion/exclusion criteria are provided below:

1. **Population:** University students (who are not necessarily always adult), including undergraduates, postgraduates, and in specialized fields (like medicine) enrolled in a college or university in an ASEAN country were considered as the population of interest. The Association of Southeast Asian Nations (ASEAN) includes ten countries: Indonesia, Malaysia, The Philippines, Singapore, Thailand, Brunei Darussalam, Vietnam, Laos, Myanmar, and Cambodia.
2. **Intervention / exposures:** Due to the nature of the study outcome and the objectives, most studies are not expected to involve a specific intervention, but instead report on exposures associated with the outcome. Exposures of primary interest, in keeping with the objectives, are socio-demographic variables, including (but not limited to) gender, age, income, parental alcohol consumption, religion and religiosity, and peer influence.
3. **Comparator:** University students in similar settings who do not consume alcohol are considered as the control or comparator group.
4. **Outcomes:** Alcohol consumption is the primary outcome of interest, but related outcomes (like binge drinking) were also compiled, where applicable or available.
5. **Study design:** The type of study was not restricted in the initial literature search, and both observational (e.g., cross-sectional, cohort) and interventional studies were considered for inclusion.
6. **Timeframe:** While recent research (1990-2024) was prioritized, the review did not limit the search by publication date, acknowledging that earlier studies may be particularly relevant in countries with limited recent studies.

**Table 1.** Quality assessment of cross-sectional studies included in this review, using the AXIS critical appraisal instrument

| Study | Q1 | 2 | 3 | 4 | 5 | 6 | 7 | 8 | 9 | 10 | 11 | 12 | 13 | 14 | 15 | 16 | 17 | 18 | 19 | 20 | Study quality |
|---|---|---|---|---|---|---|---|---|---|---|---|---|---|---|---|---|---|---|---|---|---|
| Htet et al 2020 | Y | Y | Y | Y | Y | Y | N | Y | Y | Y | Y | Y | U | N | Y | Y | Y | Y | N | Y | H |
| Jaichuen et al 2018 | Y | Y | Y | Y | Y | Y | N | Y | Y | Y | Y | Y | U | N | Y | Y | Y | Y | N | Y | H |
| Mathialagan and Teng 2017 | Y | Y | N | Y | N | N | N | Y | Y | Y | Y | Y | U | N | N | Y | Y | Y | N | Y | M |
| Buakate et al 2022 | Y | Y | Y | Y | Y | Y | N | Y | Y | Y | Y | Y | U | N | Y | Y | Y | N | N | Y | H |
| San San et al 2010 | Y | Y | Y | Y | Y | Y | N | Y | Y | Y | Y | Y | N | N | Y | Y | Y | N | N | Y | H |
| Phoosuwan 2019 | Y | Y | Y | Y | Y | Y | N | Y | Y | Y | Y | Y | U | N | Y | Y | Y | N | N | Y | H |
| Supit et al 2017 | Y | Y | N | Y | N | Y | N | Y | Y | Y | Y | Y | U | N | Y | Y | Y | N | N | Y | M |
| Aung et al 2019 | Y | Y | Y | Y | Y | Y | Y | Y | Y | Y | Y | Y | N | N | Y | Y | Y | Y | N | Y | H |
| Soket al 2020 | Y | Y | Y | Y | Y | Y | Y | Y | Y | Y | Y | Y | N | Y | Y | Y | Y | Y | N | Y | H |
| T Nguyen et al 2019 | Y | Y | N | Y | N | N | N | Y | Y | Y | Y | N | U | N | Y | Y | Y | Y | N | Y | M |
| Caffrey et al 1996 | Y | Y | N | Y | Y | Y | N | Y | Y | N | Y | Y | U | N | Y | Y | Y | Y | N | Y | H |
| Tonkuriman et al 2019 | Y | Y | N | Y | Y | Y | N | Y | Y | Y | Y | Y | U | N | Y | Y | Y | Y | N | Y | H |
| Boonchuaythanasit et al 2021 | Y | Y | N | Y | N | Y | N | Y | Y | Y | Y | Y | U | N | Y | Y | Y | Y | N | Y | H |
| Yi et al 2017 | Y | Y | N | Y | Y | Y | N | Y | Y | Y | Y | Y | N | N | Y | Y | Y | Y | N | Y | H |
| Wattanapisit, Abdul Rahman et al 2022 | Y | Y | N | Y | Y | Y | N | Y | Y | Y | Y | Y | U | N | Y | Y | Y | Y | N | Y | H |

a Question texts for each question are reported in Table 2

b Y=YES, N=NO, U=DO NOT KNOW; note that for Q19, N is considered as a positive score for the study, while for all other questions, Y is positive (encoded as 1), and both N and U are encoded as negative or potentially negative (score encoded as 0)

c For the final column of the table, a study is deemed as H: high quality (low risk of bias), M: medium quality (moderate risk of bias), and L: low quality (high risk of bias) for scores between 15-20, 10-14, and <10, respectively

**Table 2.** Texts of questions in the AXIS critical appraisal instrument

| Question ID | Relevant Section | Text |
|---|---|---|
| 1 | Introduction | Were the aims/objectives of the study clear? |
| 2 | Methods | Was the study design appropriate for the stated aim(s)? |
| 3 | | Was the sample size justified? |
| 4 | | Was the target/reference population clearly defined? (Is it clear who the research was about?) |
| 5 | | Was the sample frame taken from an appropriate population base so that it closely represented the target/reference population under investigation? |
| 6 | | Was the selection process likely to select subjects/participants that were representative of the target/reference population under investigation? |
| 7 | | Were measures undertaken to address and categorise non-responders? |
| 8 | | Were the risk factor and outcome variables measured appropriate to the aims of the study? |
| 9 | | Were the risk factor and outcome variables measured correctly using instruments/measurements that had been trialled, piloted or published previously? |
| 10 | | Is it clear what was used to determined statistical significance and/or precision estimates? (eg, p values, CIs) |
| 11 | | Were the methods (including statistical methods) sufficiently described to enable them to be repeated? |
| 12 | Results | Were the basic data adequately described? |
| 13 | | Does the response rate raise concerns about non-response bias? |
| 14 | | If appropriate, was information about non-responders described? |
| 15 | | Were the results internally consistent? |
| 16 | | Were the results for the analyses described in the methods, presented? |
| 17 | Discussion | Were the authors' discussions and conclusions justified by the results? |
| 18 | | Were the limitations of the study discussed? |
| 19 | Other | Were there any funding sources or conflicts of interest that may affect the authors' interpretation of the results? |
| 20 | | Was ethical approval or consent of participants attained? |
